# Dissemination of *bla*_KPC-3_-harbouring *Klebsiella pneumoniae* across ST48 and ST628 in multiple healthcare facilities in the Republic of Korea

**DOI:** 10.1080/22221751.2026.2691352

**Published:** 2026-09-01

**Authors:** Min Kyeong Kim, Kyeong Woo Noh, Eunkyung Shin, Jungsun Park, Ae Kyung Park, Seongjae Joo, Seo Hyun Lee, Da Eun Oh, Min Ji Kim, Min Hee Heo, Seung Yeon Jang, Junyoung Kim

**Affiliations:** a Division of Bacterial Diseases, Department of Laboratory Diagnosis and Analysis, Korea Disease Control and Prevention Agency, Cheongju-si, Republic of Korea; b Division of Infectious Disease Diagnosis, Jeonbuk State Institute of Health and Environment Research, Imsil-gun, Republic of Korea

**Keywords:** Carbapenem-resistant Enterobacterales, *Klebsiella pneumoniae*, *bla*_KPC-3_, clonal dissemination, horizontal plasmid transfer

## Abstract

*Klebsiella pneumoniae* carbapenemase-3 (KPC-3) remains rare in South Korea, where KPC-2 is the dominant carbapenemase, making the repeated detection of a concentrated *bla*_KPC-3_ signal over five years notable. We performed genomic analyses of *bla*_KPC-3_-harbouring *K. pneumoniae* from a regional healthcare network. Two chromosomally distinct lineages with concordant capsule loci (ST628/KL15 and ST48/KL62) presented multidrug-resistant phenotypes, and the virulence-associated loci were confined to ST48. Single-nucleotide polymorphism (SNP) analyses revealed near-clonal relatedness within lineages, with 0–38 pairwise SNPs among ST628 isolates and 8 SNPs between the two ST48 isolates. Core-genome multilocus sequence typing (cgMLST) supported this structure, as ST628 isolates were assigned to complex type 19149 with 0–7 allelic differences, and ST48 isolates were assigned to complex type 19150 with 5 allelic differences. These patterns support vertical spread via clonal expansion across multiple facilities. Despite substantial chromosomal separation, most isolates carried the same IncFII(K) plasmid backbone and *bla*_KPC-3_, and they were nearly indistinguishable from a plasmid previously reported in South Korea. One isolate carried *bla*_KPC-3_ on a distinct multireplicon IncFIB(K)/IncFII(K) plasmid, indicating that the signal was not confined to a single plasmid backbone. In both plasmids, *bla*_KPC-3_ was embedded within Tn*4401b*. These findings indicate that a rare *bla*_KPC-3_ genotype can persist regionally through sustained clonal dissemination and that cross-lineage linkage is compatible with past horizontal transfer involving a conserved plasmid. These findings underscore the need for subtype-resolved, regionally coordinated genomic surveillance in connected healthcare networks to detect uncommon carbapenemase variants early.

## Introduction

Nationwide surveillance of carbapenem-resistant Enterobacterales (CRE) has consistently aided in identifying *Klebsiella pneumoniae* as the predominant species and *K. pneumoniae* carbapenemase (KPC)-type carbapenemase genes as the prevailing determinants in South Korea. During the past five years, *bla*_KPC-2_ has accounted for 80% of cases of carbapenemase detection, whereas the prevalence of *bla*_KPC-3_ has remained ≤1%. These numbers align with national reports describing *K. pneumoniae* and *bla*_KPC-2_ as the leading species‒genotype combination in South Korea [1–3]. A geographically concentrated signal of *bla*_KPC-3_ is therefore notable against a background in which *bla*_KPC-3_ is rare.

Carbapenem-resistant *K. pneumoniae* (CRKP) is transmitted efficiently within and between healthcare facilities, facilitated by patient movement, invasive devices, and environmental persistence, and it imposes a substantial clinical and public-health burden [4,5]. Moreover, international experience has shown that *bla*_KPC-3_-harbouring *K. pneumoniae* can drive subsequent hospital outbreaks despite its low frequency. Reported episodes included tertiary-care ICU outbreaks in Israel in 2006 that were later linked to a national epidemic, ICU outbreaks in Italy in 2011, a hospital outbreak in Mexico City in 2013, a single-centre paediatric outbreak in the United States in 2015, and a hospital outbreak in China during 2020–2021 [6–10]. Together, these reports indicate that *bla*_KPC-3_ can sustain transmission once established in a local setting, underscoring the importance of resolving geographically concentrated signals when they arise.

At the global level, KPC-producing *K. pneumoniae* dissemination has historically been dominated by a clonal group (CG) 258 – comprising ST258, ST512, and the closely related ST11 – but genomic surveillance has since revealed an increasingly polyclonal landscape, with non-CG258 lineages such as ST307, ST147, ST48, and ST628 emerging as important *bla*_KPC_ carriers across multiple continents [11,12]. *K. pneumoniae* ST48 has been detected in clinical settings spanning Asia, Africa, and Europe, and is characterized by the presence of diverse carbapenemases including NDM-type, OXA-48-like, and KPC enzymes, with global phylogenomic analyses supporting its intercontinental dissemination [13–15]. ST628, although encountered less frequently than established high-risk clones, has been associated with nosocomial outbreaks and sporadic cases in the United Kingdom, the United States, Spain, Brazil, and India, with certain strains demonstrating the capacity to acquire carbapenemase-encoding plasmids [16,17]. In South Korea, where the KPC epidemic was established primarily through the spread of ST307 and ST11 in 2014–2015, the detection of *bla*_KPC-3_-harbouring ST48 and ST628 within a geographically concentrated healthcare network represents an epidemiological signal that warrants a detailed investigation [18].

The Korea Disease Control and Prevention Agency (KDCA) conducts molecular epidemiologic surveillance of CRE using pulsed-field gel electrophoresis (PFGE) and whole-genome sequencing (WGS). PFGE is performed on *K. pneumoniae* isolates recovered from CRE patients through the provincial public health laboratory network, and selected isolates undergo WGS. At the national scale, PFGE profiles are heterogeneous and largely concordant with the *bla*_KPC-2_-predominant background. Repeated analyses have consistently revealed a recurrent cluster confined to a single geographic area. All the isolates in this cluster were *bla*_KPC-3_-harbouring *K. pneumoniae*, whereas *bla*_KPC-3_ was only rarely detected at the national level. PFGE identified 32 *bla*_KPC-3_-harbouring *K. pneumoniae* strains detected only within that region, with pattern similarity ranging from 59.7% to 100%. The genomic analysis focused on a selected subset to investigate the recurrent regional signal across the healthcare network. The analysed isolates were collected between 2020 and 2024 from eight healthcare facilities (four general hospitals, one tertiary-care hospital, and three long-term care hospitals).

We therefore integrated epidemiological data with WGS data to examine the regional *bla*_KPC-3_ signal in a connected healthcare network. This approach allowed us to assess the relative contributions of sustained clonal dissemination and linkage through a conserved *bla*_KPC-3_ plasmid platform to the observed regional pattern.

## Materials and methods

### Selection of study isolates

PFGE identified 32 *bla*_KPC-3_-positive isolates in KDCA surveillance. Eleven of these isolates were selected for whole-genome sequencing based on two criteria: (i) PFGE pattern identity (100% Dice similarity) within a cluster and (ii) involvement of the tertiary-care hospital, which served as the regional referral hub and was consistently represented in the recurrent interfacility cluster signal. This approach was designed to capture the most epidemiologically plausible transmission events within the regional referral network. Isolates that met both criteria belonged to two PFGE-identical clusters (n = 11). The remaining 21 isolates exhibited distinct PFGE patterns or did not involve the tertiary-care hospital and were not included in the genomic analysis.

### Bacterial identification and antimicrobial susceptibility testing

Species were identified using the VITEK 2 system (bioMérieux, Marcy-l’Étoile, France) with GN identification cards. Minimum inhibitory concentrations (MICs) were determined using Sensititre KORN and KRCDC2F (customized) panels (Thermo Fisher Scientific, Waltham, MA, USA) covering 19 antimicrobial agents. Interpretive categories were assigned according to Clinical and Laboratory Standards Institute (CLSI) M100 breakpoints where available.

### DNA extraction and whole-genome sequencing

Single colonies were cultured on nutrient medium at 37°C for 16–20 h, and genomic DNA was extracted using the DNeasy Blood & Tissue Kit (Qiagen, Hilden, Germany) according to the manufacturer’s instructions. Short-read libraries were prepared with Illumina DNA Prep and sequenced on a MiSeq platform (Illumina, San Diego, CA, USA). Long-read libraries were prepared with the Native Barcoding Kit (SQK-NBD114.24) and sequenced on a MinION device using R10.4.1 flow cells (Oxford Nanopore Technologies, Oxford, UK).

### Read processing and hybrid genome assembly

Paired-end reads were processed with fastp v1.0.1 using a Q20 quality cut-off. Reads with >30% bases below Q20 or <50 nt after trimming were discarded. Long reads were filtered with Filtlong v0.3.1, retaining reads ≥1 kb and the top 90% by read quality. Hybrid assemblies were generated from the filtered short and long reads with Unicycler v0.5.0. The resulting assemblies were annotated with Bakta v1.11.4 before downstream genotypic analyses.

### Genotypic analysis and chromosomal relatedness

The genome assemblies were analysed with PathogenWatch (https://pathogen.watch/) to infer the multilocus sequence type (MLST), serotype, virulence gene content, and antimicrobial resistance determinants. For the ST-specific comparative analysis, publicly available genomes were retrieved separately for ST628 and ST48 using the BV-BRC Similar Genome Finder with KP20-314 and KP24-501 as the respective query genomes and the search scope set to all public genomes. Candidate genomes were curated manually, with priority given to complete genomes where available and with efforts to reduce the overrepresentation of highly similar genomes from the same country. The final comparative datasets comprised 20 publicly available genomes of ST628 and 23 genomes of ST48 (Table S3). Pairwise single-nucleotide polymorphism (SNP) distances were obtained using CSI Phylogeny v1.4, with sequence type (ST)-specific reference genomes. ST628 isolates were mapped to *K. pneumoniae* UDH-49_PH (Accession no. GCF_029624875.1), whereas ST48 isolates were mapped to *K. pneumoniae* NKP14 (Accession no. GCF_022029115.1). Maximum-likelihood phylogenies were reconstructed from the ST-specific SNP alignments using IQ-TREE v3.1.1, and branch support was assessed by SH-aLRT and ultrafast bootstrapping. Core-genome MLST (cgMLST) was performed using Ridom SeqSphere+ (Ridom GmbH, Münster, Germany).

### Plasmid characterization

Plasmid sequences were further characterized for antimicrobial resistance determinants, plasmid replicons, mobility, and mobile genetic elements. Antimicrobial resistance determinants and plasmid replicons were identified using ABRicate v1.0.1 against the ResFinder and PlasmidFinder databases, with minimum identity and coverage thresholds set to 90% and 80%, respectively. Mobile genetic elements were identified using MobileElementFinder v1.1.2. Plasmid mobility was assessed using the MOB suite (mob_typer v3.1.9). IncFII(K) plasmid contigs were compared with the reference plasmids pKPC_Kp46 from *K. pneumoniae* strain Kp46 (GenBank accession no. KX348146.1) and p2017-45-54-05_3 from *Enterobacter asburiae* strain 2017-45-54-05 (GenBank accession no. CP109696.1) using MUMmer4 to enumerate SNPs and insertion/deletion (indel) events. Gene synteny and the pairwise nucleotide identity of the *bla*_KPC-3_ region were visualized using clinker with representative *bla*_KPC-3_-carrying reference plasmids listed in Supplementary Table S4. A plasmid map was generated and visualized with Proksee (https://proksee.ca/).

## Results

### Antimicrobial susceptibility profiles

The results of antimicrobial susceptibility testing are summarized in Table 1. Resistance to carbapenems (imipenem, meropenem, doripenem, and ertapenem) was observed in all the isolates. Among the carbapenems tested, resistance to ertapenem was universal, whereas resistance to imipenem, meropenem, and doripenem was detected in three isolates each (27.3%). Resistance to cefoxitin, cefotaxime, ceftriaxone, and ceftazidime, as well as to ciprofloxacin and nalidixic acid, was also detected in all the isolates. Resistance to trimethoprim–sulfamethoxazole was frequent (81.8%), whereas resistance to tetracycline and chloramphenicol was less common (18.2% and 9.1%, respectively). No isolate was resistant to aminoglycosides (amikacin or gentamicin) or colistin. All the isolates were classified as multidrug resistant (MDR). No isolate was classified as extensively drug resistant (XDR) or pandrug resistant (PDR).

**Table 1.** Minimum inhibitory concentrations (MICs) of antimicrobial agents for *bla*_KPC-3_-harbouring *Klebsiella pneumoniae* isolates.

Isolate	MICs (µg/mL)																		
	β-lactams					Carbapenems				Quinolones/ Fluoroquinolones		Aminoglycosides			Other classes				
	AMP	FOX	CTX	AXO	CAZ	IPM	MEM	DOR	ETP	CIP	NAL	AMI	GEN	STR	SXT	TET	AZI	CHL	COL
KP20-314	>64	>32	>32	>32	>16	>32	>32	>32	>32	4	32	≤4	4	>128	>16/304	8	16	8	≤2
KP21-624	>64	32	>32	>32	>16	≤0.5	≤0.5	≤0.5	2	4	32	≤4	2	>128	>16/304	8	16	8	≤2
KP21-783	>64	16	>32	>32	>16	1	≤0.5	1	2	2	32	≤4	2	>128	>16/304	8	16	16	≤2
KP22-255	>64	16	>32	>32	>16	1	≤0.5	≤0.5	4	2	32	≤4	2	>128	>16/304	8	16	8	≤2
KP22-440	>64	32	>32	>32	>16	1	≤0.5	≤0.5	2	2	32	≤4	4	>128	>16/304	8	16	16	≤2
KP22-831	>64	>32	>32	>32	>16	8	16	8	32	>16	>128	≤4	2	32	≤1/19	128	4	32	≤2
KP23-738	>64	16	>32	>32	>16	≤0.5	≤0.5	≤0.5	2	2	32	≤4	4	>128	>16/304	8	16	8	≤2
KP24-501	>64	16	>32	>32	>16	4	4	4	4	>16	>128	≤4	4	32	≤1/19	128	8	4	≤2
KP24-1115	>64	16	>32	>32	>16	≤0.5	≤0.5	≤0.5	2	2	32	≤4	2	>128	>16/304	8	16	8	≤2
KP24-1146	>64	16	>32	>32	>16	≤0.5	≤0.5	≤0.5	2	4	32	≤4	2	>128	>16/304	8	16	16	≤2
KP24-1267	>64	16	>32	>32	>16	≤0.5	≤0.5	≤0.5	2	4	32	≤4	4	>128	>16/304	8	32	16	≤2

Abbreviations: AMP, ampicillin; FOX, cefoxitin; CTX, cefotaxime; AXO, ceftriaxone; CAZ, ceftazidime; IPM, imipenem; MEM, meropenem; DOR, doripenem; ETP, ertapenem; CIP, ciprofloxacin; NAL, nalidixic acid; AMI, amikacin; GEN, gentamicin; SXT, trimethoprim/sulfamethoxazole; TET, tetracycline; AZI, azithromycin; CHL, chloramphenicol; STR, streptomycin; COL, colistin.

### Genomic characteristics and typing

#### Sequence types, capsule loci, and virulence-associated loci

WGS assigned the 11 isolates to two sequence types: ST628 (n = 9) and ST48 (n = 2). Capsular locus typing was concordant within each sequence type. All the ST628 isolates were assigned as KL15, whereas both ST48 isolates were assigned as KL62.

An analysis of virulence-associated loci showed that aerobactin (*iuc1*) and salmochelin (*iro1*) were present in the two ST48 isolates, which also carried *rmp* loci consistent with a KpVP-1-like virulence-associated gene repertoire. The remaining nine ST628 isolates lacked *iuc*, *iro*, and *rmp* loci. Yersiniabactin (*ybt*) and colibactin (*clb*) were not detected in any of the isolates.

#### Antimicrobial resistance determinants

All the isolates carried multiple antimicrobial resistance determinants (Table 2). For β-lactams, every isolate harboured *bla*_KPC-3_ and *bla*_CTX-M-15_, with additional *bla*_SHV-1_ (100%), *bla*_TEM-1_ (81.8%), and *bla*_OXA-1_ (18.2%). A frameshift mutation in the outer membrane porin gene *ompK36* (D28fs) was detected in one isolate (9.1%). Aminoglycoside resistance genes included *aac(6′)-Ib* and *aadA1* in all the isolates, together with *aph(6)-Id* (81.8%), *aph(3″)-Ib* (81.8%), and *aac(6′)-Ib-cr* (18.2%). Quinolone resistance determinants included *oqxA* (100%), *oqxB* (100%), *qnrB19* (100%), and *qnrB1* (81.8%). In addition, mutations in *gyrA* (S83Y) and *parC* (S80I) were detected in both ST48 isolates (18.2%). Additional resistance determinants included *sul2* (81.8%; sulphonamides), *dfrA14* (81.8%; trimethoprim), and *tet(A)* (18.2%; tetracycline).

**Table 2.** Antimicrobial resistance genes and key mutations in *bla*_KPC-3_- harbouring *K. pneumoniae* isolates.

Isolate	Carbapenemase	β-lactamase	Aminoglycoside	Quinolone	Sulfoamide	Tetracycline	Trimethoprim	Fosfomycin	Porin mutation	QRDR mutation
KP20-314	*bla*_KPC-3_	*bla*_CTX-M-15_, *bla*_SHV-1_, *bla*_TEM-1_	*aadA1, aac(6′)-Ib, aph(3″)-Ib, aph(6)-Id,*	*qnrB1, qnrB19, oqxAB*	*sul2*	–	*dfrA14*	*fosA6*	*ompK36*: D28fs	–
KP21-624	*bla*_KPC-3_	*bla*_CTX-M-15_, *bla*_SHV-1_, *bla*_TEM-1_	*aadA1, aac(6′)-Ib, aph(3″)-Ib, aph(6)-Id*	*qnrB1, qnrB19, oqxAB*	*sul2*	–	*dfrA14*	*fosA6*	–	–
KP21-783	*bla*_KPC-3_	*bla*_CTX-M-15_, *bla*_SHV-1_, *bla*_TEM-1_	*aadA1, aac(6′)-Ib, aph(3″)-Ib, aph(6)-Id*	*qnrB1, qnrB19, oqxAB*	*sul2*	–	*dfrA14*	*fosA6*	–	–
KP22-255	*bla*_KPC-3_	*bla*_CTX-M-15_, *bla*_SHV-1_, *bla*_TEM-1_	*aadA1, aac(6′)-Ib, aph(3″)-Ib, aph(6)-Id*	*qnrB1, qnrB19, oqxAB*	*sul2*	–	*dfrA14*	*fosA6*	–	–
KP22-440	*bla*_KPC-3_	*bla*_CTX-M-15_, *bla*_SHV-1_, *bla*_TEM-1_	*aadA1, aac(6′)-Ib, aph(3″)-Ib, aph(6)-Id*	*qnrB1, qnrB19, oqxAB*	*sul2*	–	*dfrA14*	*fosA6*	–	–
KP22-831	*bla*_KPC-3_	*bla*_CTX-M-15_, *bla*_SHV-1_, *bla*_OXA-1_	*aadA1, aac(6′)-Ib, aac(6′)-Ib-cr*	*qnrB19, oqxAB*	–	*tet(A)*	–	*fosA6*	–	GyrA:S83Y ParC:S80I
KP23-738	*bla*_KPC-3_	*bla*_CTX-M-15_, *bla*_SHV-1_, *bla*_TEM-1_	*aadA1, aac(6′)-Ib, aph(3″)-Ib, aph(6)-Id*	*qnrB1, qnrB19, oqxAB*	*sul2*	–	*dfrA14*	*fosA6*	–	–
KP24-501	*bla*_KPC-3_	*bla*_CTX-M-15_, *bla*_SHV-1_, *bla*_OXA-1_	*aadA1, aac(6′)-Ib, aac(6′)-Ib-cr*	*qnrB19, oqxAB*	–	*tet(A)*	–	*fosA6*	–	GyrA:S83Y ParC:S80I
KP24-1115	*bla*_KPC-3_	*bla*_CTX-M-15_, *bla*_SHV-1_, *bla*_TEM-1_	*aadA1, aac(6′)-Ib, aph(3″)-Ib, aph(6)-Id*	*qnrB1, qnrB19, oqxAB*	*sul2*	–	*dfrA14*	*fosA6*	–	–
KP24-1146	*bla*_KPC-3_	*bla*_CTX-M-15_, *bla*_SHV-1_, *bla*_TEM-1_	*aadA1, aac(6′)-Ib, aph(3″)-Ib, aph(6)-Id*	*qnrB1, qnrB19, oqxAB*	*sul2*	–	*dfrA14*	*fosA6*	–	–
KP24-1267	*bla*_KPC-3_	*bla*_CTX-M-15_, *bla*_SHV-1_, *bla*_TEM-1_	*aadA1, aac(6′)-Ib, aph(3″)-Ib, aph(6)-Id*	*qnrB1, qnrB19, oqxAB*	*sul2*	–	*dfrA14*	*fosA6*	–	–

### Genomic relatedness and clonal structure

#### SNP-based phylogenetic analysis

**Figure 1.**
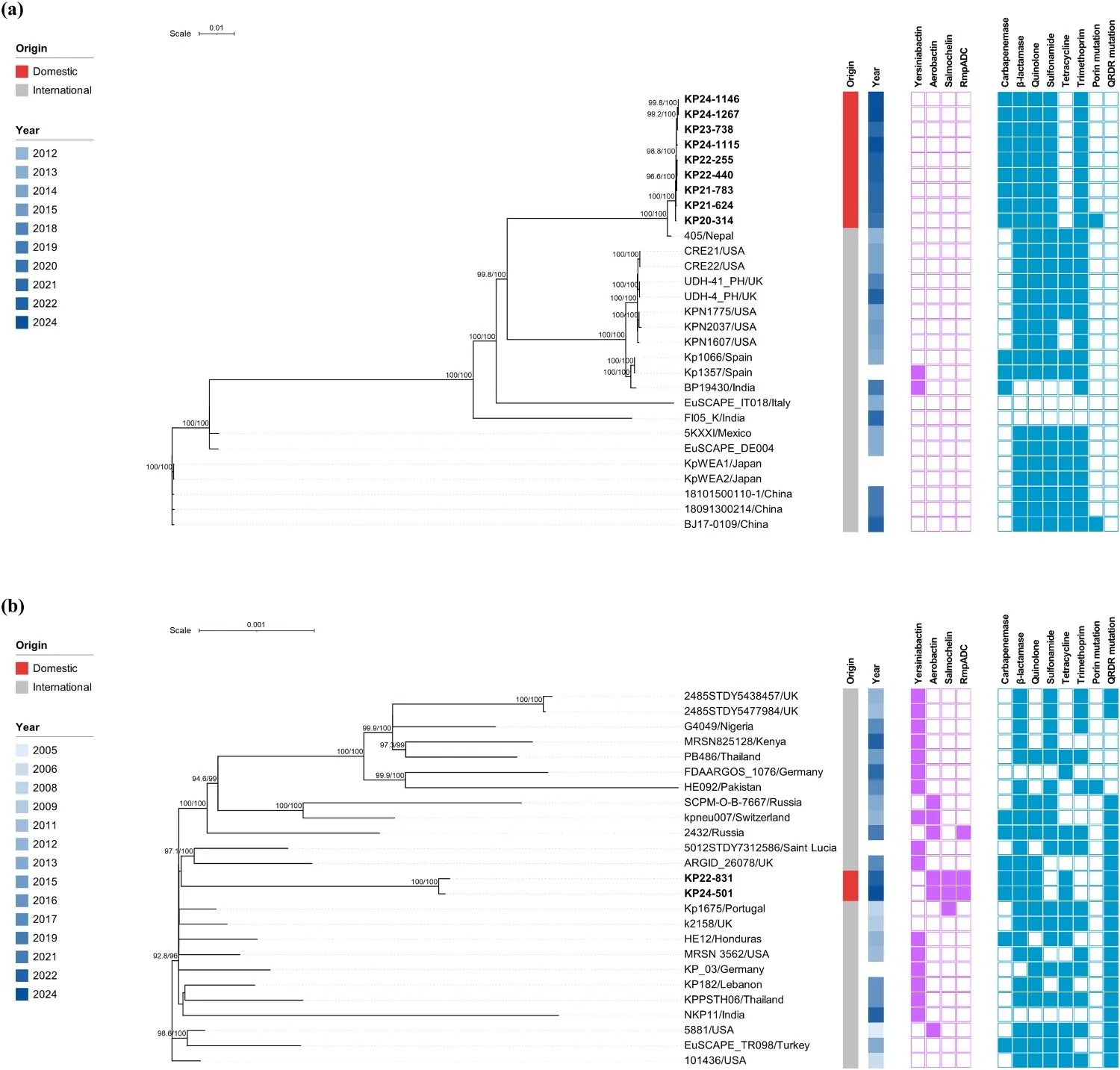
Maximum-likelihood phylogenies and associated genomic features of *bla*_KPC-3_-harbouring *K. pneumoniae*. Maximum-likelihood phylogenies of (a) ST628 and (b) ST48 isolates are shown with their genomic features. The numbers at the nodes indicate the SH-aLRT/UFBoot support values. The bars indicate the isolate origin and year of collection. The columns summarize the virulence-associated loci and antimicrobial resistance determinants. Filled boxes indicate the presence of the indicated loci, determinants, or mutations, and empty boxes indicate their absence. RmpADC denotes rmp-associated loci, and QRDR denotes the quinolone resistance-determining region. The scale bar indicates genetic distance. Isolates analysed in this study are indicated in bold.

Clonal relatedness was assessed by performing SNP-based analysis within ST-specific comparative datasets, and the results were visualized as maximum-likelihood phylogenies with branch support (Figure 1). For the ST628 isolates (n = 9), the pairwise SNP distances ranged from 0 to 38, whereas comparisons with publicly available ST628 genomes revealed broader divergence (144–5,210 SNPs); the closest genome was strain 405 (Nepal), with 144–168 SNPs. In the ST628 maximum-likelihood phylogeny, the nine Korean isolates formed a tight cluster, with internal nodes showing SH-aLRT/UFBoot values of 96.6/100–100/100 (Figure 1(a)). For the ST48 study isolates (n = 2), the two isolates differed by 8 SNPs and showed 138–351 SNPs relative to publicly available ST48 genomes; the closest genomes were strain 101436 (USA; 138–140 SNPs), strain Kp1675 (Portugal; 139–141 SNPs), and strain 5881 (USA; 140–142 SNPs). In the ST48 maximum-likelihood phylogenies, the two Korean isolates formed a close pair within the broader international ST48 background, and this pairing was supported by an SH-aLRT/UFBoot value of 100/100 (Figure 1(b)). Together, the low pairwise SNP distances and high branch support values supported close within-ST clustering of the Korean isolates.

#### cgMLST-based minimum spanning tree analysis

**Figure 2.**
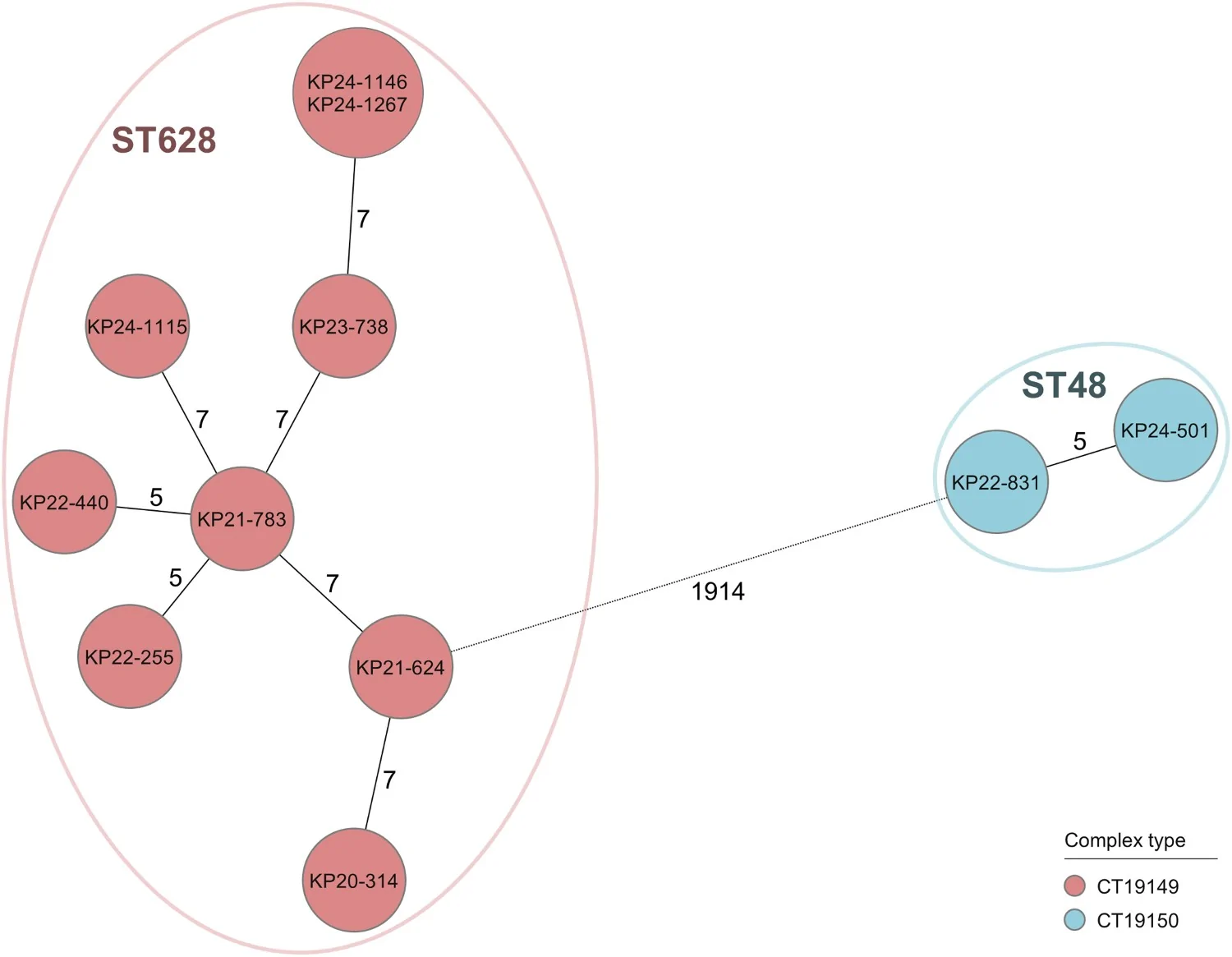
Minimum spanning tree based on the cgMLST allelic profiles of 11 *bla*_KPC-3_-carrying *K. pneumoniae* isolates. The MST was constructed from allelic profiles of the *K. pneumoniae* sensu lato cgMLST scheme, comprising 2,358 loci. Edge labels indicate pairwise allelic differences, which were calculated with missing values ignored. Nodes represent isolates and are coloured according to the complex type (CT19149 for ST628, pink; CT19150 for ST48, blue). Encircling boundaries delineate each complex type.

cgMLST supported the clustering observed in the SNP-based maximum-likelihood phylogenies (Figure 2). The nine ST628 isolates were assigned to complex type 19149. KP24-1146 and KP24-1267 were indistinguishable, and links within the ST628 cluster differed by 5–7 alleles. The two ST48 isolates belonged to complex type 19150 and differed by 5 alleles. The ST628 and ST48 clusters were widely separated by 1,914 allelic differences, which is consistent with their distinct chromosomal backgrounds.

### Plasmid architecture and bla_KPC-3_ genetic context

#### Genetic composition of bla_KPC-3_-carrying plasmids

**Figure 3.**
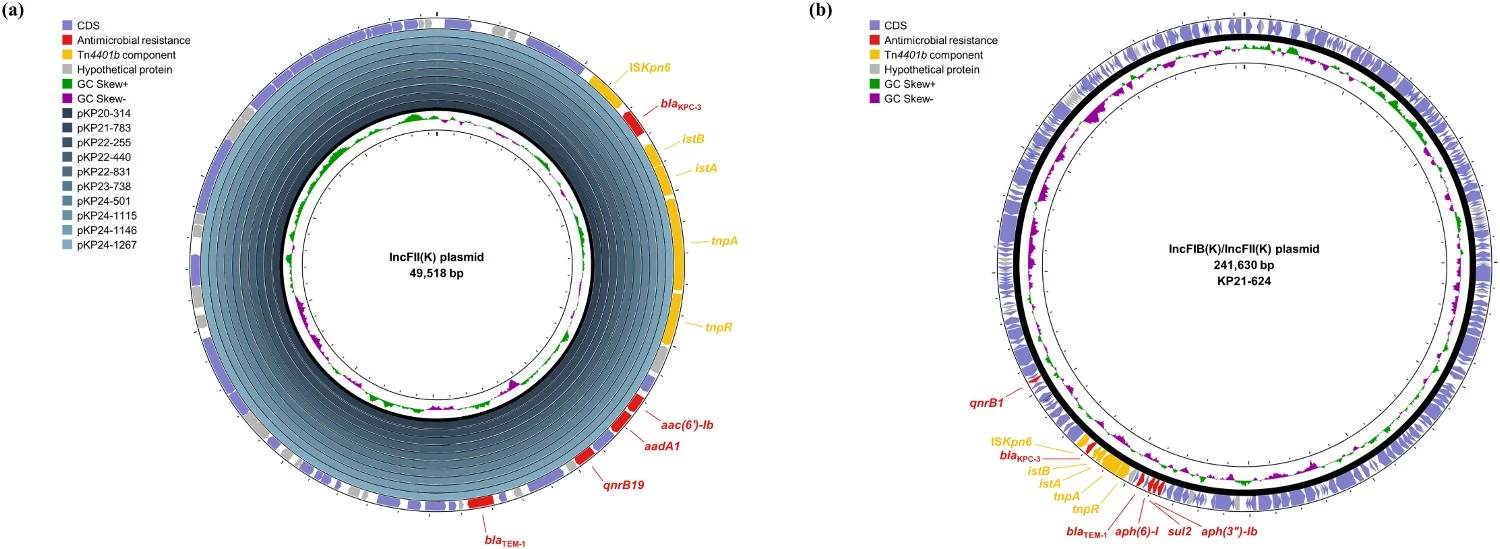
Plasmid maps of *bla*_KPC-3_-carrying plasmids in *K. pneumoniae* isolates. (a) Comparative circular map of *bla*_KPC-3_-carrying IncFII(K) plasmids from 10 isolates, showing the conserved plasmid backbone shared across most of the studied isolates. From the inside to the outside circles: (i) scale (bp), (ii) GC skew, (iii) plasmid rings for each isolate, and (iv) functionally classified gene annotations are shown. (b) Circular map of the *bla*_KPC-3_-carrying IncFIB(K)/IncFII(K) plasmid from isolate KP21-624. Genes in both panels are coloured according to the same functional categories.

We compared the *bla*_KPC-3_-carrying plasmids to determine whether the regional *bla*_KPC-3_ signal was linked to a shared mobile platform. Ten of the eleven *bla*_KPC-3_-harbouring *K. pneumoniae* isolates carried a highly conserved plasmid, regardless of the sequence type. This plasmid measured 49,518 bp, belonged to the incompatibility group IncFII(K), and showed >99.99% nucleotide sequence identity to pKPC_Kp46 (Figure 3(a) and Table S1). The backbone was also highly concordant with that of p2017-45-54-05_3 (Table S2). Within this conserved IncFII(K) plasmid, *bla*_KPC-3_ colocalized with additional resistance determinants, including *bla*_TEM-1_, *qnrB19*, *aadA1*, and *aac(6′)-Ib*, and it was accompanied by mobilization-associated functions, such as a MOB_C-family relaxase and a VirB4-like ATPase.

The remaining isolate carried *bla*_KPC-3_ on a 241,630 bp multireplicon IncF plasmid harbouring IncFIB(K) and IncFII(K) replicons, the backbone of which corresponded to that of pKp591-231 (GenBank accession no. CP028817.1) (Figure 3(b)). The alignment covered 95.85% of the plasmid, and the aligned regions showed 99.97% nucleotide identity. In addition to carrying *bla*_KPC-3_, this plasmid carried *bla*_TEM-1_, *qnrB1*, *sul2*, *dfrA14*, *aph(6)-Id*, and *aph(3″)-Ib* and encoded proteins with conjugation-associated functions, including a MOBF-type relaxase, a VirD4-family type IV coupling protein, and a VirB4-family ATPase.

#### Conserved Tn4401b context and flanking repeats

**Figure 4.**
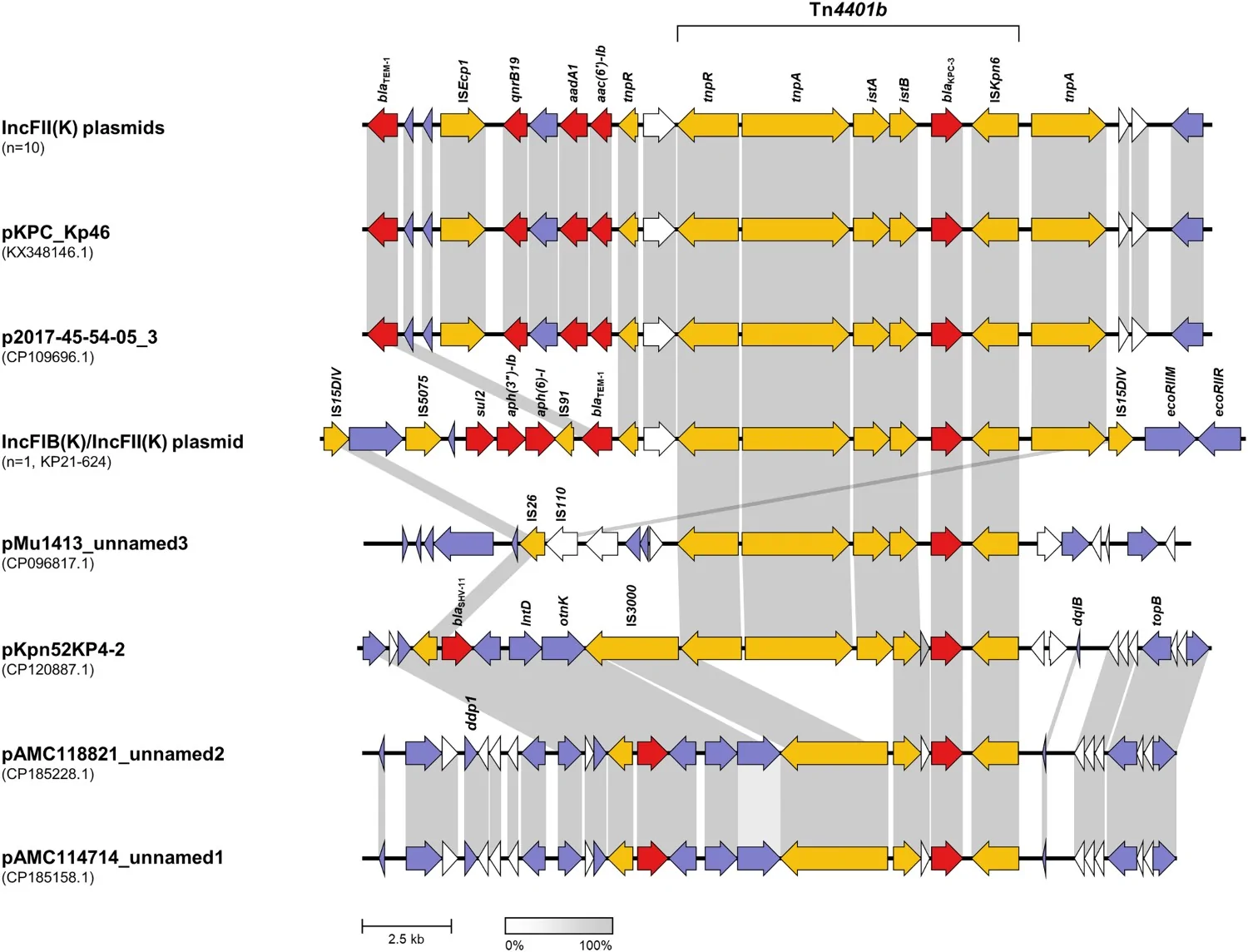
Structural comparison of the *bla*_KPC-3_ genetic environment between the plasmids identified in this study and reference plasmids. The *bla*_KPC-3_ locus and adjacent regions were aligned to compare the Tn*4401b*-associated genetic structures of the IncFII(K) plasmids identified in this study and the *bla*_KPC-3_-carrying reference plasmids pKPC_Kp46, p2017-45-54-05_3, pMu1413_unnamed3, pKpn52KP4-2, pAMC118821_unnamed2, and pAMC114714_unnamed1. The uppermost row represents the shared *bla*_KPC-3_/Tn*4401b*-associated arrangement observed in the 10 IncFII(K) plasmids identified in this study. The GenBank accession numbers for the reference plasmids are shown below each plasmid name. The arrows indicate predicted coding sequences and transcriptional orientation. The bracket denotes the Tn*4401b* region carrying *bla*_KPC-3_. The shaded blocks between sequences represent nucleotide similarity, and the scale bar corresponds to 2.5 kb. Details of the reference plasmids used for the structural comparison are provided in Supplementary Table S4.

The genetic context of *bla*_KPC-3_ was conserved across both plasmid backbones. In both the IncFII(K) and IncFIB(K)/IncFII(K) plasmids, *bla*_KPC-3_ was embedded within Tn*4401b* with the arrangement *tnpR*-*tnpA*-IS*Kpn7*-*bla*_KPC-3_-IS*Kpn6*. The terminal inverted repeats were flanked by identical 5 bp direct repeats (GTTCT). In the ten IncFII(K) plasmids, the coding DNA sequence (CDS) of the surrounding region was identical and showed 100% nucleotide identity to that of pKPC_Kp46 and p2017-45-54-05_3 across the aligned segment (Figure 4). In contrast, the previously reported domestic *bla*_KPC-3_ plasmids pAMC118821_unnamed2 and pAMC114714_unnamed1 showed different gene organization in the corresponding region and lacked the intact Tn*4401* structure observed in our isolates.

## Discussion

CRE is listed in a critical priority group, the highest tier of the 2024 World Health Organization (WHO) bacterial priority pathogens list [19]. In South Korea, CRE infection is currently managed as a class 2 notifiable infectious disease. However, between 2016 and 2019, CRE infection was managed as a class 4 notifiable infectious disease under sentinel surveillance, and nationwide isolate retrieval for a molecular epidemiological analysis was not yet feasible. As the national CRE burden increased, mandatory full notification and nationwide molecular surveillance were subsequently established [20]. The national number of CRE infections increased from 23,311 in 2021 to 42,347 in 2024. The proportion of carbapenemase-producing Enterobacterales (CPE) also increased from 61.9% in 2020 to 78.3% in 2024, supporting the growing contribution of carbapenemase-mediated resistance to the national CRE burden. These trends may reflect not only expanded detection but also structural changes in healthcare delivery. An expanding population of older and immunocompromised patients, longer lengths of stay, and frequent transfers between long-term care hospitals and acute-care facilities create repeated opportunities for CRE to be maintained and amplified across connected facilities [20,21]. In this context, healthcare-associated CRE infection is typically shaped by a few dominant carbapenemase lineages. Therefore, determining whether the regional *bla*_KPC-3_ signal follows the same pattern is important. Assessing whether this uncommon subtype reflects only clonal spread or a combination of clonal spread and linkage through a shared mobile genetic platform is also important.

National and regional reports have described healthcare-associated dissemination dominated by common carbapenemase backgrounds, including KPC-producing *K. pneumoniae,* in hospital outbreaks [18,22]. In this setting, repeated detection of *bla*_KPC-3_ across multiple facilities within a single region is notable. These findings suggest that uncommon subtypes may also be maintained once they are introduced into a highly connected care environment. This pattern has practical implications for surveillance. When monitoring remains limited to broad genotype categories or focuses mainly on historically dominant lineages, early signals from rare subtypes may be delayed or missed, which is especially relevant when transmission is distributed across multiple facilities rather than concentrated within a single ward. These findings support the use of subtype-resolved, regionally coordinated molecular surveillance in healthcare networks with ongoing CRE transmission [18,20,22]. Additionally, interpreting early warning signals requires sustained clonal spread to be distinguished from cross-lineage dissemination within the same regional network. In this context, the recovery of closely related *bla*_KPC-3_-harbouring isolates across multiple facility types over several years is more compatible with regional maintenance within a connected healthcare network than with isolated, facility-restricted events. Within the regional referral architecture, where the tertiary-care hospital functions as the principal acute-care hub for surrounding general hospitals and long-term care facilities, the distribution of closely related isolates across eight facilities provides a plausible structural pathway for interfacility spread. The observed genomic pattern is therefore more consistent with referral network-mediated dissemination than with independent facility-level outbreaks, although a direct epidemiological linkage between individual cases could not be confirmed in the present study. The facility type distribution across the regional network is compatible with patient transfer between acute and long-term care settings as a contributing mechanism of interfacility spread, which is consistent with established CRE transmission pathways in connected healthcare systems. Whether individual clusters within facilities represent localized outbreaks or continued importation from the regional referral network could not be determined from the genomic data alone and warrants further epidemiological investigation with complete patient-level contact data.

Chromosomal relatedness provides the most direct evidence for clonal dissemination. In the ST-specific maximum-likelihood phylogenies, the Korean isolates clustered within each sequence type rather than being dispersed across broader international backgrounds, and this topology was supported by high branch support values. Together with the low within-ST SNP distances and cgMLST clustering, three converging lines of genomic evidence support this interpretation. The Korean ST628 isolates formed a tight monophyletic cluster in the maximum-likelihood phylogeny (SH-aLRT/UFBoot: 96.6/100–100/100), with within-Korea pairwise SNP distances of 0–38 SNPs – more than an order of magnitude lower than the distance to the nearest publicly available ST628 genome (144–168 SNPs; strain 405, Nepal). The same pattern held for ST48: the two Korean isolates differed by 8 SNPs from each other but by 138–140 SNPs from the nearest international genome (strain 101436, USA). cgMLST further assigned all the Korean isolates within each ST to a single complex type (CT19149 for ST628 and CT19150 for ST48), with a maximum intracluster allelic distance of 7 alleles across isolates from eight facilities over four years. The magnitude of the gap between within-Korea diversity and the distance to any known international genome, together with the single complex type assignment, is inconsistent with repeated recent independent introductions and instead supports descent from a locally established common ancestor. This interpretation is also supported by concordant capsular types and largely conserved AMR and virulence-associated gene content within each ST. The persistence of closely related isolates across multiple facilities over several years is compatible with local establishment within the regional healthcare network. This pattern is less consistent with repeated independent introductions into individual facilities. *bla*_KPC-3_ was detected in both ST628 and ST48, indicating that the regional signal was not restricted to a single host background. The ST48 background carried canonical virulence-associated loci, including aerobactin and salmochelin, suggesting the presence of a virulence-associated genetic repertoire that may be relevant to persistence or colonization in healthcare settings; as these inferences are based on genomic detection alone, their clinical significance requires experimental confirmation [23,24]. The isolates were MDR, which can limit therapeutic options and contribute to poor clinical outcomes [25].

Taken together, these findings support sustained clonal expansion as the primary mode of regional dissemination. Moreover, the presence of a highly conserved *bla*_KPC-3_ plasmid backbone across distinct lineages is compatible with at least one past horizontal transfer event. The shared IncFII(K) plasmid was nearly indistinguishable from a plasmid reported in South Korea in 2015 and remained closely related to an international *bla*_KPC-3_-carrying reference plasmid, indicating that the regional signal was linked to a shared mobile platform rather than to one chromosomal lineage alone. This similarity suggests possible long-term maintenance of this IncFII(K) lineage in South Korea. However, a direct molecular comparison across the intervening period was not feasible because, during 2016–2019, CRE infection in South Korea was managed under sentinel surveillance, which did not permit nationwide isolate retrieval or a systematic molecular epidemiological analysis. These findings should therefore be interpreted as compatible with possible long-term maintenance rather than as definitive evidence of continuous persistence.

One isolate instead carried *bla*_KPC-3_ on a distinct IncFIB(K)/IncFII(K) plasmid, indicating that *bla*_KPC-3_ within this regional network was not confined to a single plasmid backbone. Although conjugation assays were not performed, *in silico* mobility typing and structural annotation revealed transfer-associated features in both plasmid types. The occurrence of related *bla*_KPC-3_ platforms in two distinct chromosomal backgrounds (ST628 and ST48) separated by 1,914 cgMLST allelic differences is compatible with at least one past horizontal transfer event, although this result remains inferential. The conserved Tn*4401b* structure across both backbones further supports linkage through a shared *bla*_KPC-3_ genetic platform [26,27].

Together, these findings support a layered model in which sustained clonal expansion drives the dominant regional signal, while conserved *bla*_KPC-3_-carrying plasmid platforms may intermittently link distinct host backgrounds. This pattern suggests that the regional *bla*_KPC-3_ signal was shaped by both clonal spread and mobile genetic linkage [28,29].

This analysis focused on genomic relatedness and plasmid context in a selected subset of isolates and did not cover the full PFGE diversity of all 32 *bla*_KPC-3_-positive isolates. The findings should therefore be interpreted in the context of the study design. Detailed epidemiological data on patient movement were not available for all cases; thus, transmission was inferred mainly from genomic relatedness rather than from complete contact networks. In addition, a direct molecular comparison with isolates circulating between 2015 and 2020 was not feasible because nationwide isolate retrieval and molecular surveillance were not yet established during the earlier sentinel-surveillance phase in South Korea. Most importantly, this study relied entirely on genomic and *in silico* analyses, and no wet-lab experimental validation was performed. Plasmid mobility was inferred from structural features and *in silico* mobility typing rather than confirmed by conjugation assays, and the virulence-associated potential of the ST48 isolates was inferred from the genomic detection of virulence-associated loci rather than validated by phenotypic or *in vivo* functional assays. Consequently, our conclusions regarding horizontal plasmid transfer potential and virulence-associated characteristics remain strictly inferential and should not be over-interpreted as evidence of experimentally confirmed plasmid transfer or clinically confirmed virulence. Functional studies, including *in vitro* conjugation experiments and phenotypic or animal-model virulence assays, will be required to substantiate these genomic inferences. Finally, the scarcity of comparable *bla*_KPC-3_-carrying plasmids in public repositories constrained the breadth of plasmid comparisons. Despite these constraints, the combined chromosomal and plasmid analyses provide a useful genomic framework for interpreting this uncommon regional *bla*_KPC-3_ signal. These findings also support the use of sustained, subtype-resolved genomic surveillance for detecting both ongoing clonal spread and occasional cross-lineage linkage in connected healthcare networks.

## Conclusions

These findings support a regional genomic epidemiological interpretation of *bla*_KPC-3_ dissemination in South Korea. Sustained clonal expansion across facilities appeared to be the dominant mode of spread, while the detection of related *bla*_KPC-3_-carrying plasmids in distinct chromosomal backgrounds remained compatible with occasional cross-lineage linkage. Repeated detection of this rare subtype across multiple facilities underscores the need for subtype-resolved, regionally coordinated genomic surveillance. Integrating chromosomal relatedness with the mobile genetic context may improve the early detection of uncommon carbapenemase variants and support more targeted regional infection-control interventions in connected healthcare networks.

## Supplementary Material

KPC3Kp_Supplementary_Table_revised-clean.docx

## Data Availability

The draft genome assemblies of all 11 isolates analysed in this study have been deposited in the National Center for Biotechnology Information (NCBI) GenBank under BioProject PRJNA1418923. The corresponding BioSamples accession numbers are SAMN55067164–SAMN55067174, and the assembly accession numbers range from JBUESB000000000 to JBUESL000000000.
