# Supplementary material for: Dissemination of blaKPC-3-harbouring Klebsiella pneumoniae across ST48 and ST628 in multiple healthcare facilities in the Republic of Korea
Source: Emerg Microbes Infect. 2026 Sep 1;15(1):2691352. doi: 10.1080/22221751.2026.2691352 (PMC13537060; doi:10.1080/22221751.2026.2691352)
Supplement: Supplementary file 1 [file TEMI_A_2691352_SM0029.docx]

Supplementary Table S1. Sequence differences in *bla*_KPC-3_-carrying IncFII(K) plasmids from the study isolates relative to the reference plasmid pKPC_Kp46.

| **Isolate** | **Identity (%)** | **Indel** |  |  | **SNPs** |  |  |
| --- | --- | --- | --- | --- | --- | --- | --- |
|  |  | **Position** | **Ref** | **Alt** | **Position** | **Ref** | **Alt** |
| KP20-314 | 99.996 | 44,089 | - | A | 6,140 | C | A |
|  |  |  |  |  | 25,279 | C | T |
| KP21-783 | 100 | 44,089 | - | A | - | - | - |
| KP22-255 | 99.998 | 44,089 | - | A | 6,140 | C | A |
| KP22-440 | 99.994 | 44,089 | - | A | 6,140 | C | A |
|  |  |  |  |  | 6,308 | G | A |
|  |  |  |  |  | 25,279 | C | T |
| KP22-831 | 99.998 | 44,089 | - | A | 21,384 | A | G |
| KP23-738 | 99.998 | 44,089 | - | A | 25,279 | C | T |
| KP24-501 | 99.994 | 44,089 | - | A | 12,198 | G | A |
|  |  |  |  |  | 12,244 | A | G |
|  |  |  |  |  | 25,279 | C | T |
| KP24-1115 | 99.998 | 44,089 | - | A | 25,279 | C | T |
| KP24-1146 | 99.992 | 44,089 | - | A | 6,140 | C | A |
|  |  |  |  |  | 6,308 | G | A |
|  |  |  |  |  | 25,279 | C | T |
|  |  |  |  |  | 44,847 | A | T |
| KP24-1267 | 99.996 | 44,089 | - | A | 25,279 | C | T |
|  |  |  |  |  | 44,847 | A | T |

Supplementary Table S2. Sequence differences in *bla*_KPC-3_-carrying IncFII(K) plasmids from the study isolates relative to the reference plasmid p2017-45-54-05_3.

| **Isolate** | **Identity (%)** | **Indel** |  |  | **SNPs** |  |  |
| --- | --- | --- | --- | --- | --- | --- | --- |
|  |  | **Position** | **Ref** | **Alt** | **Position** | **Ref** | **Alt** |
| KP20-314 | 99.992 | - | - | - | 11,984 | C | T |
|  |  | - | - | - | 25,778 | A | C |
|  |  | - | - | - | 27,639 | C | A |
|  |  | - | - | - | 46,778 | C | T |
| KP21-783 | 99.996 | - | - | - | 11,984 | C | T |
|  |  | - | - | - | 25,778 | A | C |
| KP22-255 | 99.994 | - | - | - | 11,984 | C | T |
|  |  | - | - | - | 25,778 | A | C |
|  |  | - | - | - | 27,639 | C | A |
| KP22-440 | 99.990 | - | - | - | 11,984 | C | T |
|  |  | - | - | - | 25,778 | A | C |
|  |  | - | - | - | 27,639 | C | A |
|  |  | - | - | - | 27,807 | G | A |
|  |  | - | - | - | 46,778 | C | T |
| KP22-831 | 99.994 | - | - | - | 11,984 | C | T |
|  |  | - | - | - | 25,778 | A | C |
|  |  | - | - | - | 42,883 | A | G |
| KP23-738 | 99.994 | - | - | - | 11,984 | C | T |
|  |  | - | - | - | 25,778 | A | C |
|  |  | - | - | - | 46,778 | C | T |
| KP24-501 | 99.990 | - | - | - | 11,984 | C | T |
|  |  | - | - | - | 25,778 | A | C |
|  |  | - | - | - | 33,697 | G | A |
|  |  | - | - | - | 33,743 | A | G |
|  |  | - | - | - | 46,778 | C | T |
| KP24-1115 | 99.994 | - | - | - | 11,984 | C | T |
|  |  | - | - | - | 25,778 | A | C |
|  |  | - | - | - | 46,778 | C | T |
| KP24-1146 | 99.988 | - | - | - | 11,984 | C | T |
|  |  | - | - | - | 16,829 | A | T |
|  |  | - | - | - | 25,778 | A | C |
|  |  | - | - | - | 27,639 | C | A |
|  |  | - | - | - | 27,807 | G | A |
|  |  | - | - | - | 46,778 | C | T |
| KP24-1267 | 99.992 | - | - | - | 11,984 | C | T |
|  |  | - | - | - | 16,829 | A | T |
|  |  | - | - | - | 25,778 | A | C |
|  |  | - | - | - | 46,778 | C | T |

Supplementary Table S3. Publicly available reference genomes used for the ST-specific comparative genomic analyses in this study.

| **MLST** | **Strain** | **Country** | **Year** | **Assembly accession** |
| --- | --- | --- | --- | --- |
| ST628 | 405 | Nepal | 2012 | GCA_022244655.1 |
|  | 5KXXI | Mexico | 2013 | GCF_018137745.1 |
|  | EuSCAPE_DE004 | Germany | 2013 | GCF_900512385.1 |
|  | EuSCAPE_IT018 | Italy | 2013 | GCF_900514155.1 |
|  | Kp1066 | Spain | 2013 | GCF_003226115.1 |
|  | CRE21 | USA | 2014 | GCF_003400895.1 |
|  | CRE22 | USA | 2014 | GCF_003400915.1 |
|  | Kp1357 | Spain | 2014 | GCF_003226075.1 |
|  | KPN1607 | USA | 2014 | GCA_022080095.1 |
|  | KPN1775 | USA | 2014 | GCA_022082395.1 |
|  | KPN2037 | USA | 2015 | GCA_022061565.1 |
|  | UDH-41_PH | United Kingdom | 2018 | GCF_029624935.1 |
|  | 18091300214 | China | 2019 | GCF_018274905.1 |
|  | 18101500110-1 | China | 2019 | GCF_018275085.1 |
|  | BP19430 | India | 2019 | GCF_011742315.1 |
|  | FI05_K | India | 2021 | GCF_029521405.1 |
|  | BJ17-0109 | China | 2022 | GCF_023700585.1 |
|  | UDH-4_PH | United Kingdom | 2022 | GCF_029624895.1 |
|  | KpWEA1 | Japan | - | GCF_021655435.1 |
|  | KpWEA2 | Japan | - | GCF_021655455.1 |
| ST48 | 5881 | USA | 2005 | GCF_030845955.1 |
|  | 101436 | USA | 2006 | GCF_001647455.1 |
|  | Kp1675 | Portugal | 2008 | GCF_903171865.1 |
|  | k2158 | United Kingdom | 2009 | GCF_900085965.1 |
|  | 2485STDY5477984 | United Kingdom | 2011 | GCA_900560465.1 |
|  | KP_03 | Germany | 2011 | GCF_026240595.1 |
|  | MRSN 3562 | USA | 2011 | GCF_000982215.1 |
|  | 2485STDY5438457 | United Kingdom | 2012 | GCA_900560705.1 |
|  | HE12 | Honduras | 2012 | GCF_000982085.1 |
|  | kpneu007 | Switzerland | 2012 | GCF_900607495.1 |
|  | EuSCAPE_TR098 | Turkey | 2013 | GCF_900506445.1 |
|  | SCPM-O-B-7667 | Russia | 2013 | GCF_015645885.1 |
|  | PB486 | Thailand | 2015 | GCF_900093145.1 |
|  | KP182 | Lebanon | 2016 | GCF_022818905.1 |
|  | KPPSTH06 | Thailand | 2016 | GCF_002249125.1 |
|  | ARGID_26078 | United Kingdom | 2017 | GCF_922829425.1 |
|  | G4049 | Nigeria | 2017 | GCF_002187995.1 |
|  | HE092 | Pakistan | 2017 | GCF_900182165.1 |
|  | 2432 | Russia | 2019 | GCF_011044735.1 |
|  | FDAARGOS_1076 | Germany | 2021 | GCF_016727425.1 |
|  | MRSN825128 | Kenya | 2022 | GCF_024990435.1 |
|  | NKP11 | India | 2022 | GCF_022029055.1 |
|  | 5012STDY7312586 | Saint Lucia | - | GCF_900774865.1 |

Supplementary Table S4. Reference plasmids used for the structural comparison of the *bla*_KPC-3_/Tn*4401b* region.

| **Plasmid** | **Bacterial host** | **Country** | **Year** | **GenBank accession** |
| --- | --- | --- | --- | --- |
| pKPC_Kp46 | *Klebsiella pneumoniae* strain Kp46 | Republic of Korea | 2015 | KX348146.1 |
| p2017-45-54-05_3 | *Enterobacter asburiae* strain 2017-45-54-05 | USA | 2017 | CP109696.1 |
| pMu1413_unnamed3 | *Klebsiella pneumoniae* strain Mu1413 | USA | 2015 | CP096817.1 |
| pKpn52KP4-2 | *Klebsiella pneumoniae* strain GIMC1005:Kpn52KP4 | Russia | 2021 | CP120887.1 |
| pAMC118821_unnamed2 | *Klebsiella pneumoniae* strain AMC118821 | Republic of Korea | 2021 | CP185228.1 |
| pAMC114714_unnamed1 | *Klebsiella pneumoniae* strain AMC114714 | Republic of Korea | 2021 | CP185158.1 |
